# A “one stop liver shop” approach improves the cascade-of-care for Aboriginal and Torres Strait Islander Australians living with chronic hepatitis B in the Northern Territory of Australia: results of a novel care delivery model

**DOI:** 10.1186/s12939-020-01180-w

**Published:** 2020-05-07

**Authors:** Thel K. Hla, Sarah M. Bukulatjpi, Paula Binks, George G. Gurruwiwi, Roslyn G. Dhurrkay, Jane Davies

**Affiliations:** 1 Royal Darwin Hospital, Rocklands Drive, Tiwi, Darwin, NT 0810 Australia; 2 Menzies School of Health Research, Charles Darwin University, Rocklands Drive, Tiwi, Darwin, NT 0810 Australia; 3 Menzies School of Health Research, Rocklands Drive, Tiwi, Darwin, NT 0810 Australia

**Keywords:** Chronic hepatitis B, Aboriginal Australians, Cascade of care, Novel care delivery

## Abstract

**Background:**

Aboriginal and Torres Strait Islander Australians are disproportionately affected by Chronic Hepatitis B (CHB) with a prevalence of 6.08% in the Northern Territory (NT) and liver cancer rates 6 times higher than non-Indigenous Australians. Without appropriate care, overall 25% of those living with CHB will die from either liver failure or liver cancer, outcomes that can be minimised with regular follow up, antiviral treatment and hepatocellular carcinoma (HCC) screening. This care including antiviral treatment is publicly funded in the Australian setting however the care cascade still shows inequities in access to treatment for Aboriginal Australians. We describe the impact of a mobile care delivery model, “One Stop Liver Shop”, on the cascade of care for people living with CHB in a remote Australian setting.

**Methods:**

A retrospective analysis was performed for CHB care received between 2013 and 2018 in one very remote Northern Territory community, where the “One Stop Liver Shop” was iteratively developed with the community. Patients with positive Hepatitis B virus surface antigen (HBsAg) were identified through electronic medical records. Proportions of patients who are up-to-date with monitoring investigations and HCC screening were evaluated and compared to national guidelines and targets.

**Results:**

Eighty-three HBsAg positive patients were evaluated. Eighty-eight percent were engaged in care, 16% of whom were receiving antiviral treatment. Liver function tests (LFT) were up to date in 71% of patients in 2013 and 88% in 2018. Viral load (VL) monitoring was up to date for 61 (73%) of patients. There were 44 patients in whom HCC screening was indicated. Of these, 38 (86.4%) were up to date with 6 monthly alpha-fetoprotein (AFP), 35 (79.5%) were up to date with 6 monthly liver ultrasound scan (USS), and 34 (77.3%) were up-to-date for both.

**Conclusions:**

A “One Stop Liver Shop” developed with and including Aboriginal Health Practitioners bridges gaps in the availability of services to those living with CHB in a very remote community and improves the cascade of care.

## Introduction

The Northern Territory (NT) has the highest prevalence of chronic hepatitis B infection (CHB) in Australia at 1.90%, compared to the national average of 0.95% [1]. There is a disproportionate burden of CHB in the Aboriginal population of the NT with Hepatitis B Virus Surface Antigen (HBsAg) positivity significantly higher (6.08%) compared to the Non-Indigenous population (1.56%) [2]. Without appropriate management and timely treatment an estimated 15% of women and 40% of men living with CHB acquired early in life will die from decompensated liver cirrhosis or HCC. These outcomes can be prevented with publicly funded treatments from the Pharmaceutical Benefits Scheme (PBS), Australia’s national drug subsidy program [3–5]. Aboriginal and Torres Strait Islander peoples (hereafter respectfully referred to as Aboriginal Australians) are more likely to have limited access to appropriate testing, monitoring, treatment and care [6, 7].

In the NT, universal childhood vaccination for HBV was introduced in 1988 for Aboriginal children, as a priority group with documented high prevalence, and then expanded to include all children born since 1990. As the majority of those living with CHB acquired it at birth or in early life [8], the Aboriginal population born before 1988 continue to be at risk of CHB and are in an age group where their life-long infection increases their risk of cirrhosis and liver cancer. Australia and New Zealand have signed up to the WHO target of elimination of CHB as a public health problem by 2030 [9]. Achievement of this target will only be feasible through continued vaccination of non-immune adults and children, along with identification and provision of appropriate clinical care for those currently living with CHB (majority of whom were born prior to availability of HBV vaccination) so as to remove risk of ongoing transmission and minimise morbidity.

The recently published Third National Hepatitis B Strategy by Australian Department of Health (which highlights Aboriginal and Torres Strait Islander Peoples as a priority group) sets clear targets that 80% of infected people should be diagnosed, 50% engaged in care and 20% of those with CHB should be receiving antiviral treatment by 2022 [7]. Currently, gaps still exist in the NT with an estimated 61% of people living with CHB aware of their diagnosis, 20.5% engaged in care and only 5.2% receiving antiviral therapy [1].

Over the last 7 years, we have iteratively developed the “One Stop Liver Shop” in conjunction with one specific *very remote* community (as defined by the Australian Bureau of Statistics based on measures of relative access to services) [10] clinic. This community is over 500 km away or 2 h’ flight from Darwin, where the NT’s only tertiary hospital is located. The “One Stop Liver Shop” team is made up of a specialist doctor, a community based Aboriginal Health Practitioner (AHP) who co-ordinates the visit and patients, a sonographer and a clinical nurse specialist. The team brings along a portable ultrasound scan (USS), a transient elastography (FibroScan®) and mobile devices for education using ‘the Hep B Story app’ – which is a purpose-made mobile application designed to provide CHB related education in the patient’s first language, Yolngu Matha or English [11]. Two community based educators, as well as the co-ordinating AHPs, have been trained and supported to deliver the education using this app in a culturally safe and respectful way. Without the facilities made available by the “One Stop Liver Shop”, the nearest facility for a FibroScan® is the Royal Darwin Hospital. The nearest health facility with an USS capability is a District Hospital, which is 128 km away in direct distance but without direct flights or road access. Blood tests performed in this very remote community are processed by a private pathology provider based in a different state; the turnaround time for most tests is several days due to logistics and viral load results typically take 2–3 weeks. The “One Stop Liver Shop” visits occur 4 times per year for 2 days’ duration equating to 16 clinical sessions per annum. We examined the impact of this novel care delivery model with regards to the CHB care cascade in this community through the five-year period from 2013 to 2018, compared to targets set by the national strategy, and against Territory and national averages.

## Methods

This was a retrospective analysis of CHB related care received by residents of one specific remote community between September 2013 and September 2018. Inclusion criteria was defined as currently residing in the community and showing evidence of HBsAg positivity. No age limits were applied in this analysis. Patients were excluded if they no longer met criteria for CHB (seroconversion from HBsAg positive to HBsAg negative) or were deceased prior to the review date.

Communicare (© Telstra Health), the electronic medical record system used by the community medical clinic, was interrogated to identify all individuals utilising the services of the “One Stop Liver Shop” for CHB care over the five-year period (2013–2018). Additional patients were identified through auditing the existing referral database maintained by clinicians from the “One Stop Liver Shop” team. Demographic information regarding each patient was extracted from Communicare (name, date of birth and gender) along with results of investigations [liver function tests (LFT), HBV serology, viral load (VL), co-infection with Human Immunodeficiency Virus (HIV), Hepatitis C Virus (HCV), Hepatitis A Virus (HAV) immune status, serum alpha-fetoprotein (AFP), FibroScan® score and liver USS results]. Electronic clinical correspondence in the form of specialist clinic letters was also reviewed for information regarding the current stage of CHB and treatment status.

The frequency and timing of investigations done were assessed for timeliness according to surveillance targets recommended by The Gastroenterology Society of Australia (GESA) Chronic Hepatitis Recommendations which are as follows – at least 12 monthly LFT and VL for those in immune tolerance or immune control phase; at least 6 monthly LFT and annual VL for those in immune clearance or escape phase, and at least 6 monthly LFTs and VL for those currently controlled on antiviral treatment [12]. In accordance with guidelines from Central Australian Rural Practitioners Association (CARPA) and Australasian Society for HIV Viral Hepatitis and Sexual Health Medicine (ASHM) [13–15], Aboriginal patients aged 50 years and over were also assessed with regards to timeliness of HCC screening with at least 6 monthly USS and AFP measurements.

For the purpose of assessing the timeliness of investigations, the review date was assigned as 17th September 2018. Allowing for logistical and geographical challenges, CHB care was considered “up-to-date” if the investigations occurred within 2 months of the “due” date. Data analysis was performed using an electronic spreadsheet (Excel,©Microsoft) to calculate percentages of patients meeting “up to date” criteria outlined above. Ethical approval was obtained from the Human Research Ethics Committee of the Northern Territory Department of Health and Menzies School of Health Research (HREC 2017–2907).

## Results

A total of 98 patients with current HBsAg positivity were identified and their records were reviewed. Eleven patients no longer fitting CHB criteria (HBsAg clearance during the surveillance period) and four deceased patients were excluded, leaving 83 patients in the final analysis.

Characteristics of the included patients are summarised in Table 1. All but one patient were adults (age range 9–76 years) and all but one identified as Aboriginal Australians. The nine-year-old had been vaccinated as per the universal infant vaccination schedule in place at time of birth. There were no patients identified who were co-infected with HIV or HCV, although HIV serology status was unknown in four patients (4.8%) and HCV status was unknown in seven (8.4%).

**Table 1:** Characteristics of those living with CHB (*n* = 83)

Median age	49 years	(IQR 40–59.5)
Male	48	(58%)
eAg status		
Positive	9	(10.8%)
Negative	68	(81.9%)
Unknown	6	(7.2%)
eAb status		
Positive	59	(71.1%)
Negative	17	(20.4%)
Unknown	7	(8.4%)
Currently receiving nucleos(t) ide analogue treatment for HBV	13	(15.6%)
Co-infection		
HIV positive	0	(0%)
HCV positive	0	(0%)
HAV Immune	63	(75.9%)

Values are in numbers and (percentage) unless otherwise stated. *CHB* Chronic hepatitis B infection; eAg, e-antigen; eAb, e-antibody, *HAV* Hepatitis A virus, *HBV* Hepatitis B virus, *HCV* Hepatitis C virus, *HIV* Human Immunodeficiency virus, *IQR* Interquartile range

**Fig. 1:**
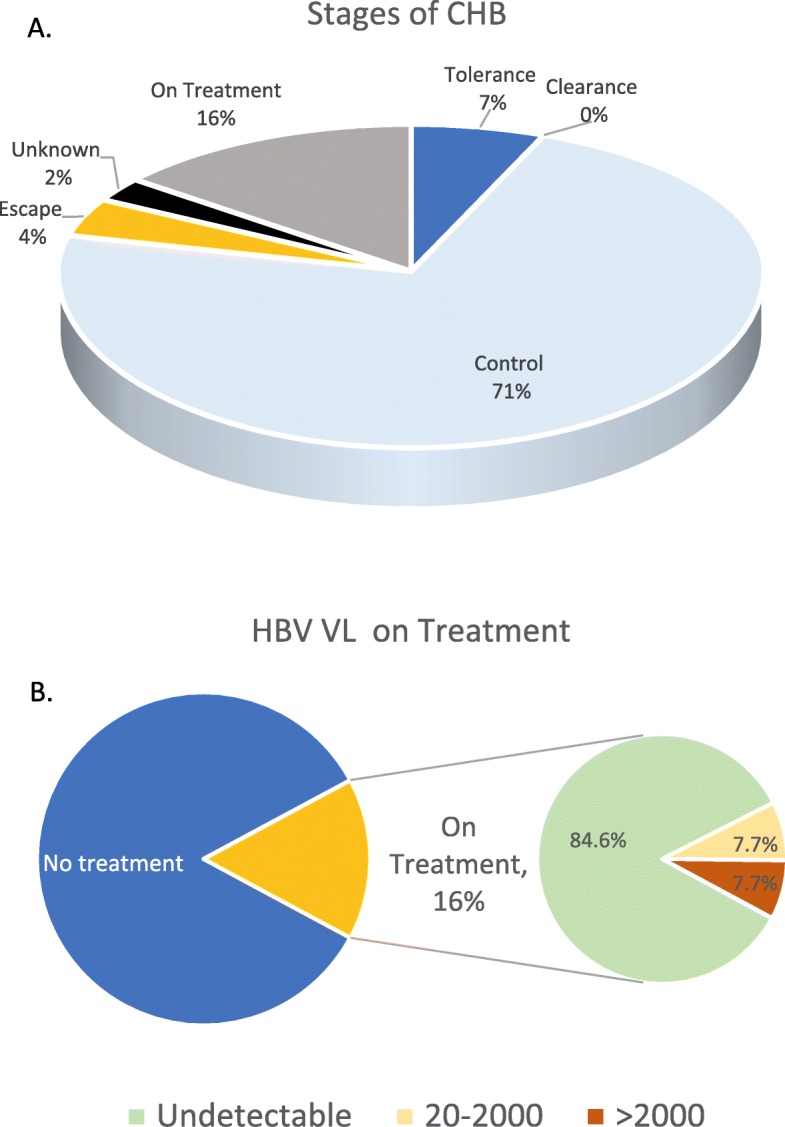
**a*.*** Distribution of CHB stages at time of study. **b*.*** Most recent viral load results for those receiving nucleos(t) ide analogue treatment. CHB, chronic hepatitis B infection; HBV, Hepatitis B Virus; VL, viral load

Distribution of patients across the various stages of CHB is represented in Fig. 1a. The median ages of patients in immune tolerance, control and escape phases were 33, 49 and 62 years respectively. There were no patients assessed to be in immune clearance phase. All three patients assessed to be in immune escape phase were due to commence treatment at the next follow up according to the specialist correspondence.

VL response for those on treatment is represented in Fig. 1b. The median age of patients on antiviral therapy was 53 years. Two out of 13 patients (15.4%) on nucleos(t) ide treatment had detectable viral loads. One was documented to be chronically non-adherent and the other had recently commenced antiviral treatment in the preceding 6 months.

**Fig. 2:**
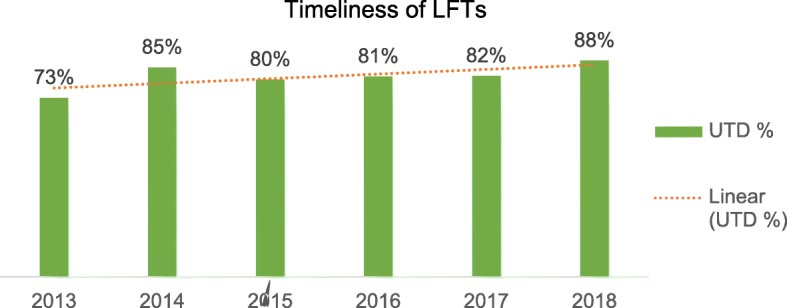
Proportion of LFT performed within the recommended period and the trends across the five-year audit period. LFT, liver function test; UTD, up-to-date

Proportion of patients meeting LFT screening guidelines for each respective CHB phase are represented in Fig. 2. Trends are plotted linearly, showing a steady increase in proportion of patients meeting surveillance targets since 2013, up to 88% in 2018. VL monitoring was up to date for 61 (73%) of patients.

There were 44 patients aged over 50 in whom HCC screening was indicated. Of these, 38 (86.4%) were up to date with 6 monthly AFP, 35 (79.5%) with 6 monthly liver USS, and 34 (77.3%) for both. Overall, 72 (86.7%) of all patients reviewed had at least one AFP measurement and 78 (93.9%) had at least one USS, of which 44 (56.4%) had at least one abnormal finding [diffuse increased echogenicity (53.8%) or evidence of portal hypertension (2.6%)].

## Discussion

This study identified and reviewed the cascade-of-care for 83 people living with CHB in one very remote NT community (population size 2206 according to the 2016 national census) [16]. Based on contemporary data which found 6.08% Aboriginal adults in this region to be HBsAg sero-positive, we estimate that there are approximately 134 people in this community living with CHB [17]. This equates to 61.9% of those living with CHB in this community being aware of their diagnosis which is in keeping with state and national figures [7, 18]. There were no cases of co-infection found with HIV or HCV which is also consistent with previous findings in this particular population [2].

The Third National Hepatitis B strategy has outlined targets that by the end of 2022, 80% of those living with CHB should be diagnosed, 50% should receive care and 20% should receive antiviral treatment [7]. Nationally, at the end of 2017, 63.7% of those living with CHB are thought to be diagnosed, while 20.2% were engaged in care with 8.3% receiving antiviral treatment [1]. In the NT, the proportion engaged in care was above the national average at 20.5%, while the proportion on treatment was below average at 5.2% [1]. In this particular remote NT community we found 88% of those aware of their CHB diagnosis were engaged in care (54.4% of estimated total) and 15.7% on treatment (9.7% of estimated total), which is well above the state and national averages. The majority of those on treatment had continued engagement and ongoing viral suppression. Therefore, the specialist outreach “One Stop Liver Shop” model has been effective in removing the barriers usually faced by residents of remote communities in terms of access to specialist clinicians and monitoring facilities such as USS or FibroScan®.

The NT has consistently had the highest prevalence of CHB since the first Hepatitis B National Mapping Report was produced using Medicare data from 2011 [19]. When engagement in care and treatment uptake were evaluated, the NT consistently had a higher proportion of individuals engaged in care. Conversely treatment uptake remained well below national averages and those set by successive National Hepatitis B strategies [1, 7, 18, 20, 21]. Between 2012 and 2017, treatment uptake improved from 2.4 to 5.2% [1, 18, 20, 21]. However, this remains below the 15 to 20% treatment targets set out by the last two national strategies [7, 22]. This likely reflects the NT’s substantial burden of CHB among rural and remote residents who are subjected to broader healthcare inequities such as, lack of access to healthcare and education, exposure to discrimination and poorer health literacy leading to inability to successfully negotiate the healthcare system [23]. The traditional confinement of CHB treatment to specialist medical practitioners and gaps in CHB knowledge in non-specialist clinicians further exacerbate the lack of access to CHB treatment and care [23].

There are multiple well documented barriers to successfully implementing CHB care including: logistics, remoteness, language, problems achieving shared understandings in a cross cultural context and low health literacy around CHB on the part of both patients and health care providers [24–28].

The holistic approach of the ‘One Stop Shop’ model is effective however its applicability is dependent on many factors such as access to diagnostic equipment, trained medical staff (GP prescribers, Nurses and AHP’s), community based educators and educational resources in local language. This may not be feasible in every remote setting. The transition of CHB care into a primary care chronic conditions model is an ongoing aspiration articulated in the NT 2014 Hepatitis B action plan [29]. The Third National HBV Strategy also highlights the need to shift the monitoring, management and care of CHB to primary care and acknowledges that this change may require the development of innovative models of care.

The success of the “One Stop Liver Shop” model provides proof of concept that a community based team approach can be effective in meeting surveillance targets for CHB. Admittedly our model had visiting specialist clinicians whereas most remote communities would only have access to general practitioners (GPs). Nevertheless, the exposure to dealing with CHB care cascade provided by the “One Stop Liver Shop” can increase competence in the local GPs and when augmented with further education, could act to support them to manage CHB locally with specialist input on request. This may already be occurring in many parts of Australia with evidence suggesting an increasing proportion of antiviral medications are prescribed by GPs for individuals living with CHB [1]. Between 2014 and 2017, GP prescriptions rose from 5.3 to 10.7% of all prescriptions [1]. Certainly, CHB care being delivered in primary care using a chronic disease model would be particularly beneficial to those dwelling in remote communities such as our study population.

The substantial contribution made by the local Aboriginal Health Practitioners (AHP) and community based educators into the overall improvements in the cascade of care in this community should also be emphasized. This is particularly significant with regards to the important role they play in delivering culturally appropriate education in first language, venepuncture in advance of the clinical visit and ensuring ongoing support and engagement. AHPs often provide continuity of care in a setting where interpersonal relationships underpin the administration of culturally safe primary care. However, training and retention of AHPs remain a major challenge in rural and remote NT where increased funding targeted at boosting the number of AHPs in the workforce has been found to be only transiently successful [30]. The AHP workforce has suffered from high rates of fading over time, often reflecting the many challenges AHPs face such as balancing various community and work responsibilities and the mismatch of training against community expectations [30]. If we are to reach and diagnose the remaining people living with CHB, it is vital that economic and logistic, as well as training and support provisions are made to ensure that AHPs remain an integral part of the care workforce.

A disproportionate burden of CHB is a common challenge facing Indigenous people in many countries, with a higher HBsAg prevalence well described in the Maori people of New Zealand, the Alaskan Inuit people and the Native American people of North America and the First Nations people of Canada [3, 31, 32]. There is scant data with regards to CHB specific cascade of care in these Indigenous populations. The cascade-of-care is better studied in those living with HIV where it is consistently demonstrated that fewer Indigenous people are engaged or retained in care compared to Non-Indigenous people, and Indigenous ethnicity is associated with an increased likelihood of suboptimal care and attrition at each stage within the HIV cascade-of-care [33, 34].

Our study was limited by its retrospective nature. We were also limited by the non-systematic method we used to identify all those living with CHB in this community, though we utilised all available records and multiple resources. We were only able to include those who were already diagnosed, and therefore unable to comment on the exact number of patients that remain to be diagnosed, though we estimate that this is likely comparable to proportions reported nationally. The future direction in this area of research is to identify all individuals living with CHB as an urgent priority as many are clinically asymptomatic with significant risk of developing complications. As the next step, and part of a recently commenced National Health and Medical Research Council (NHMRC) partnership grant, we plan to establish a HBV clinical register, the “Hep B Hub”. We will systematically assign a serology status to all residents of this community so that we can find the estimated 51 people who are unaware of their infection, vaccinate those who are non-immune and continue to monitor improvements in the cascade-of-care. Concurrently, we plan to train and maintain a competent cohort of primary healthcare professionals, including AHPs, to provide the gold standard CHB care. Through S100 prescriber training, GPs will be qualified to prescribe antivirals allowing CHB care to be successfully and sustainably transitioned into primary care. Ultimately this would benefit patients residing in remote and regional communities such as our study population.

## Conclusion

A novel, holistic multidisciplinary model of care, delivered in community can lead to significant improvements in the cascade-of-care for remote dwelling Aboriginal Australians living with CHB. Emphasis must be placed on the importance of the Aboriginal Health Practitioner’s role at the centre of the model. For this care model to be sustainable systematic implementation is essential.

## Data Availability

Due to the personal and confidential nature of the data collected a condition of our ethics approval was the raw data not be shared with any third parties. The Hep B Story App which is the bilingual electronic educational tool developed for and used in the Liver One Stop Shop is freely available from the Google Play Store, the Apple App Store and online at: https://www.menzies.edu.au/hepbstory/
